# Impact of Body Weight and Combined *UGT1A1* and *NR1I2* Polymorphisms on Dolutegravir Pharmacokinetics Among Thai People Living with HIV: A Population Pharmacokinetic Analysis

**DOI:** 10.3390/pharmaceutics18101245

**Published:** 2026-10-01

**Authors:** Anan Chanruang, Angela K. Birnbaum, Sílvia M. Illamola, Sasithorn Sirilun, Suthunya Chupradit, Sasiwimol Ubolyam, Napon Hiranburana, Yong-Soon Cho, Jae-Gook Shin, Anchalee Avihingsanon, Baralee Punyawudho

**Affiliations:** 1 PhD Degree Program in Pharmacy, Faculty of Pharmacy, Chiang Mai University, Chiang Mai 50200, Thailand; anan_c@cmu.ac.th; 2 Department of Experimental and Clinical Pharmacology, College of Pharmacy, University of Minnesota, Minneapolis, MN 55455, USA; birnb002@umn.edu (A.K.B.); sillamol@umn.edu (S.M.I.); 3 Department of Pharmaceutical Sciences, Faculty of Pharmacy, Chiang Mai University, Chiang Mai 50200, Thailand; sasithorn.s@cmu.ac.th; 4 Department of Pharmaceutical Care, Faculty of Pharmacy, Chiang Mai University, Chiang Mai 50200, Thailand; suthunya.chu@cmu.ac.th; 5 The HIV Netherlands Australia Thailand Research Collaboration (HIV-NAT), Thai Red Cross AIDS Research and Infectious Diseases Centre, Bangkok 10330, Thailand; sasiwimol.u@hivnat.org (S.U.); napon.h@hivnat.org (N.H.); anchalee.a@hivnat.org (A.A.); 6 Center for Personalized Precision Medicine of Tuberculosis, Inje University College of Medicine, Busan 47392, Republic of Korea; ysncho@gmail.com (Y.-S.C.); phshinjg@gmail.com (J.-G.S.); 7 Center of Excellence in Tuberculosis, Faculty of Medicine, Chulalongkorn University, Bangkok 10330, Thailand

**Keywords:** dolutegravir, population pharmacokinetics, Pharmacogenetics, dose optimization, Thai people living with HIV

## Abstract

**Background/Objectives**: Despite its role as a first-line therapy for Thai people living with HIV (PLWH), dolutegravir (DTG) exhibits significant pharmacokinetic (PK) variability influenced by both genetic and non-genetic factors. This variability may contribute to an increased incidence of neuropsychiatric adverse events (NP-AEs) at the standard 50 mg once daily (OD) dose. **Methods**: To evaluate the impact of relevant covariates, a population PK model of DTG was developed using 1122 DTG plasma concentrations from 221 Thai PLWH. Monte Carlo simulations were performed to evaluate the probability of target attainment for pre-defined efficacy and safety thresholds across different dosage regimens. **Results**: Data were analyzed using a one-compartment model with first-order absorption incorporating lag-time, and first-order elimination. Body weight (<61 kg) and a multigenic interaction were identified as significant covariates impacting DTG apparent clearance (CL/F). Specifically, individuals carrying both the UGT1A1 poor metabolizer phenotype and the *NR1I2* 63396 TT genotype resulted in a 25.1% reduction in DTG CL/F. Simulation results indicated that the standard dose achieved efficacy targets in > 95% of the population but resulted in a substantial proportion (30.46–85.68%) exceeding the safety threshold. In contrast, a reduced dose of 25 mg OD maintained high efficacy target attainment (>80%) while decreasing the proportion exceeding the safety threshold to <20% in most subgroups. **Conclusions**: These findings suggest that a reduced DTG dose may be a suitable alternative for Thai PLWH to mitigate the risk of NP-AEs, particularly among individuals with low body weight carrying the combined *UGT1A1* and *NR1I2* variants.

## 1. Introduction

Dolutegravir (DTG), a second-generation integrase strand transfer inhibitor (INSTI), is widely recommended as a first-line treatment for people living with HIV (PLWH), including in the 2021/2022 Thai National Guidelines [1,2]. Despite its superior efficacy and potency in viral suppression, concerns have emerged regarding a higher incidence of neuropsychiatric adverse events (NP-AEs) in real-world settings compared to clinical trial environments [3,4,5,6]. Elevated DTG plasma concentrations have been associated with an increased risk of NP-AEs, potentially leading to treatment discontinuation [4]. Furthermore, interindividual pharmacokinetic variability in a real-world cohort is substantial, with a coefficient of variation (CV) reaching up to 85%, significantly higher than the approximately 25% observed in pre-licensing trials [7,8]. Predicting individual exposure is complicated by a complex interplay of non-genetic factors, such as body weight (BW), and pharmacogenetic determinants involving key metabolic and transport pathways [9,10,11,12,13,14,15].

DTG is primarily metabolized by uridine diphosphate glucuronosyltransferase 1A1 (UGT1A1) and, to a lesser extent, by cytochrome P450 (CYP3A4) [16]. Consequently, genetic polymorphisms in *UGT1A1* significantly influence DTG pharmacokinetics and exposure. Individuals carrying UGT*1A1* intermediate or poor metabolizer alleles (e.g., *6, *28, and *37) have been shown to experience altered pharmacokinetics of integrase inhibitors, including DTG, cabotegravir, and raltegravir [8,17,18,19,20]. Beyond primary metabolism, dolutegravir (DTG) is a substrate of the efflux transporter, breast cancer resistance protein (BCRP, encoded by *ABCG2*) [16]. Furthermore, DTG disposition is modulated by the pregnane X receptor (PXR; *NR1I2*), which regulates the expression of key metabolic enzymes, including CYP3A4 and UGT1A1 [21]. Although, *ABCG2* 421C>A and *NR1I2* 63396 C>T polymorphisms have been individually reported to influence DTG plasma exposure [8,22], single-gene association studies cannot fully account for multi-pathway synergy or quantify unexplained parameter variability [8].

Physiological factors including BW significantly modulate apparent clearance and distribution volume [9,12,13,14,15]. In lower average BW populations, such as Asian cohorts, standard 50 mg once-daily dosing may lead to higher DTG exposures, increasing the risk of concentration-dependent toxicities such as NP-AEs. We therefore hypothesized that lower BW and combined pharmacogenetic variants in DTG metabolic and regulatory pathways (specifically *UGT1A1* and *NR1I2*) synergistically decrease DTG apparent clearance (CL/F), resulting in elevated plasma concentrations that may exceed established safety thresholds under standard dosing. We further hypothesized that a dose reduction to 25 mg once daily may maintain therapeutic exposure while significantly lowering the risk of exceeding the neuropsychiatric toxicity threshold in high-risk Thai PLWH. Consequently, incorporating BW and combined genetic variation into a population pharmacokinetic model is important to refine individual exposure predictions and establish evidence-based, individualized dosing strategies.

While prior statistical association studies have confirmed that pharmacogenetic variations influence the pharmacokinetics of DTG, a quantitative model that integrates both BW and multigenic interaction profiles to guide clinical dose optimization in Thai PLWH is lacking. Model-informed dose adjustment represents a promising therapeutic approach to minimize concentration-dependent toxicities while maintaining viral suppression in Thai population. To address this gap, this study presents population PK modeling and simulation analysis of DTG in a real-world Thai cohort. Using a non-linear mixed-effects modeling approach (NONMEM), we aimed to characterize population PK parameters, evaluate the combined impact of BW and multigenic profiles on DTG clearance, and perform Monte Carlo simulations to establish model-based dose optimization strategies that balance efficacy and safety in Thai PLWH.

## 2. Materials and Methods

### 2.1. Study Population and Blood Collection

This was a sub-pharmacokinetic study from two clinical trials conducted in Thai PLWH at the HIV Netherlands Australia Thailand Collaboration (HIV-NAT), Thai Red Cross AIDS and Infectious Diseases Research Centre in Bangkok, Thailand (ClinicalTrials.gov registration no. NCT03785106, 24 December 2018 and NCT03727152, 1 November 2018).

The NCT03785106 study was a multicenter, randomized, open-label, phase III clinical trial comparing a 4-week daily isoniazid/rifapentine regimen (1HP) with a 12-weekly isoniazid/rifapentine regimen (3HP) for the treatment of latent tuberculosis infection (LTBI) in PLWH without active TB. Participants received oral antiretroviral therapy (ART) consisting of DTG 50 mg combined with tenofovir alafenamide (TAF) or tenofovir disoproxil fumarate (TDF) plus emtricitabine (FTC) or lamivudine (3TC). All participants had been receiving their DTG-based ART regimen for at least 12 months prior to trial enrollment, ensuring that pharmacokinetic steady state had been achieved prior to blood sampling. For the PK sub-study (52 participants), blood samples were collected at pre-dose and 1, 2, 4, 6, 8, 10, 12, and 24 h post-dose during weeks 0 and 4, or between weeks 4 and 12, depending on the LTBI treatment regimen. Only PK data collected at week 0, prior to the initiation of LTBI therapy, were included in this analysis.

The NCT03727152 study was a phase III, open-label, single-arm study of virologically suppressed PLWH aged 18 and older. All participants previously receiving a protease inhibitor/ritonavir-based regimen were switched to a generic single-tablet regimen of TAF/FTC/DTG (50 mg of DTG). At weeks 24 and 48, intensive blood sampling was performed at pre-dose and at 1, 2, 4, 6, 8, 10, 12, and 24 h post-dose in 52 participants. Additionally, two sparse blood samples were obtained per visit from 117 participants at weeks 24 and 48, following three sampling windows: C1 (<3 h post-dose), C2 (3–5 h post-dose), or C3 (5–24 h post-dose). Demographic and laboratory data, including age, sex, BW, serum creatinine, liver function (ALT), and total bilirubin were recorded. The dosage and timing of the last administration prior to sampling, as well as the exact time of blood collection, were recorded. The study was approval by the Institutional Review Board of the Faculty of Medicine, Chulalongkorn University, Bangkok, Thailand, and the Institutional Review Board Committee on Human Research at the Faculty of Pharmacy, Chiang Mai University, Chiang Mai, Thailand.

### 2.2. Determination of DTG Plasma Concentrations

Blood samples were processed within 1 h of collection to obtain plasma and were stored at −20 °C at the HIV-NAT laboratory. Samples were subsequently shipped on dry ice to the Center for Personalized Precision Medicine of Tuberculosis, Inje University College of Medicine (Busan, Republic of Korea) for DTG concentration analysis. DTG plasma concentrations were quantified utilizing a validated liquid chromatography-tandem mass spectrometry (LC-MS/MS) method. The intraday and interday precision values were within 15%, while the accuracy ranged from 83.5% to 118.4%. The lower limit of quantification (LLOQ) was 0.1 mg/L.

### 2.3. Genotyping Analysis

Genomic DNA was extracted from peripheral blood mononuclear cells using the QIAamp DNA Blood Mini Kit (Qiagen, Hilden, Germany). The concentration and purity of the extracted DNA were assessed using a NanoDrop (Thermo Fisher Scientific, Wilmington, DE, USA). The *UGT1A1* promoter region genetic variations were amplified via polymerase chain reaction (PCR), followed by Sanger sequencing of the amplified fragments. Data were analyzed using Sequence Scanner Software v2.0 (Thermo Fisher Scientific, USA). Genotyping for the single nucleotide polymorphisms (SNPs) of *UGT1A1* 211 G>A (*6; rs4148323), *ABCG2* 421 C>A (rs2231142), and *NR1I2* 63396 C>T (rs2472677) was performed using TaqMan allelic discrimination assays on a QuantStudio 7 Flex Real-Time PCR System (Applied Biosystems, Foster City, CA, USA). The primers and PCR conditions were utilized as previously described [23].

*UGT1A1* polymorphisms were classified into three phenotype groups based on CPIC recommendations [24]: extensive metabolizers (*1/*1, *1/*36, *36/*36), intermediate metabolizers (*1/*28, *1/*37, *1/*6, *36/*28, *36/*37), and poor metabolizers (*28/*28, *28/*37, *37/*37, *6/*6). For *ABCG2* 421 C>A (rs2231142) and *NR1I2* 63396 C>T (rs2472677), genotypes were categorized into three groups: homozygous wild-type (CC), heterozygous variant (CA or CT), and homozygous variant (AA or TT). Furthermore, to evaluate potential synergistic effects, combination genotypes were classified into four distinct groups: Group 1 (UGT*1A1* poor metabolizer + *ABCG2* 421 AA + *NR1I2* 63396 TT), Group 2 (UGT*1A1* poor metabolizer + *ABCG2* 421 AA), Group 3 (UGT*1A1* poor metabolizer + *NR1I2* 63396 TT), and Group 4 (*ABCG2* 421 AA + *NR1I2* 63396 TT).

### 2.4. Population Pharmacokinetic Modeling and Statistical Analysis

Steady-state DTG pharmacokinetics were analyzed using a nonlinear mixed-effects modeling approach with NONMEM (version 7.5; Icon Development Solutions, Ellicott City, MD, USA), along with Perl-speaks-NONMEM (PsN version 5.4.0) and Pirana (version 2.8.1). The first-order conditional estimation method with interaction was employed to estimate the pharmacokinetic parameters throughout the modeling process. Graphical analysis and model evaluation were conducted using R (version 4.4.2; R Development Core Team; www.r-project.org) and Xpose package (version 4.5.0). A one-compartment model with first-order absorption and elimination was used to characterize the pharmacokinetic properties of DTG. Given that previous studies have indicated delayed absorption for DTG, both lag-time and transit compartment models were investigated. Allometric scaling based on BW was incorporated to account for the influence of body size on drug disposition, with exponents fixed at 0.75 for CL/F and 1 for apparent volume of distribution (V/F). Inter-individual variability (IIV) and inter-occasion variability (IOV) were described using an exponential model, assuming a log-normal distribution. IOV was evaluated either by incorporating it alongside IIV or by replacing IIV on absorption parameters (bioavailability; F and absorption rate constant; Ka; and lag time; ALAG). Residual unexplained variability (RUV) was evaluated using proportional, additive, and combined error models. The structural model was selected based on multiple criteria; the lowest objective function value (OFV), Akaike information criterion (AIC), successful convergence, goodness-of-fit plots (GOF), and parameter estimation precision (%RSE).

Potential covariates influencing DTG pharmacokinetic parameters, including age, sex, UGT*1A1* phenotypes, *ABCG2* 421 C>A (rs2231142), *NR1I2* 63396 C>T (rs2472677) polymorphisms, and combination genotypes (Group1, Group2, Group3, and Group 4) were investigated using a stepwise approach with forward inclusion followed by backward elimination. Continuous covariates were evaluated using linear, exponential, and power models. Categorical covariates were tested using additive and fractional models. During forward inclusion, a decrease in OFV of ≥3.84 (χ^2^ distribution; *p* < 0.05, df = 1) was required for a covariate to be added into the model. In the backward elimination, an increase in OFV of ≥6.63 (χ^2^ distribution; *p* < 0.01, df = 1) was used as the criterion for retaining a covariate in the final model.

### 2.5. Model Evaluation

Visual predictive check (VPC) and bootstrapping were conducted to assess the predictive performance of the final model and reliability of the final parameter estimates [25,26]. For the VPC, 1000 simulated concentrations were generated from the final model using the original dataset as a template. The 95% confidence intervals (CIs) of the simulated medians, as well as the 5th and 95th percentiles, were plotted against the corresponding observed data. For bootstrapping, 1000 resampled datasets were generated by random sampling with replacement from the original data. The bootstrap 95% CIs (obtained from the 2.5th and 97.5th percentiles) were generated and then compared with the estimates from the final NONMEM model.

### 2.6. Simulations for Dose Optimization

To explore the influence of significant covariates on DTG exposure and investigate the optimal dosing strategy for Thai PLWH, Monte Carlo simulations were performed using the final population pharmacokinetic model. Steady-state trough concentrations (C_trough_) were simulated under various dosing scenarios, including DTG 50 mg once daily (OD) and DTG 25 mg OD to optimize dosing regimens. These simulations were conducted using R (version 4.4.2; R Foundation for Statistical Computing) with the mrgsolve package (version 1.6.1).

For each dosage regimen, 10,000 DTG C_trough_ were simulated for each covariate category: low-weight (≤61.0 kg) and high-weight (>61.0 kg) groups, as well as individuals carrying or not carrying the Group 3 variant genotypes (UGT*1A1* poor metabolizer and *NR1I2* 63396 TT). The Group 3 variant genotypes were assigned to 1 (carriers) or 0 (non-carriers). Virtual individual BW were generated via random sampling from uniform distributions bounded by the empirical ranges observed in the study cohort: 36.8 to 61.0 kg for the low-weight group (≤61.0 kg) and 61.1 to 115.1 kg for the high-weight group (>61.0 kg). Steady-state C_trough_ values were sampled at 24 h post-dose.

The simulated DTG C_trough_ for each dosage regimen were compared based on the percentage of PLWH achieving efficacy thresholds, specifically the in vitro protein-adjusted 90% inhibitory concentration (IC90; >0.064 mg/L) and the in vivo 90% effective concentration (EC90; >0.3 mg/L) [7,27,28]. Furthermore, the proportion of simulated C_trough_ values exceeding the safety threshold of 1.34 mg/L, a level previously identified as significantly associated with NP-AE, was evaluated [29].

## 3. Results

This analysis included 1124 DTG plasma concentrations from 221 Thai PLWH. Two observed DTG concentrations were below the LLOQ and were consequently excluded from the population pharmacokinetic analysis [30]. Participant characteristics and the distribution of genetic polymorphisms are summarized in Table 1. The variant allele frequencies were 26.24% for *ABCG2* 421A, 60.63% for *NR1I2* 63396T, and 7.01% for UGT1A1*6 (211A). For, the *UGT1A1* (TA)_n_ promoter repeat, the frequencies of the TA5, TA6, TA7, and TA8 were 0.23%, 75.34%, 22.85%, and 1.58%, respectively. All observed frequencies were in accordance with the Hardy–Weinberg equilibrium (*p* > 0.05).

### 3.1. Population Pharmacokinetics Analysis

**Figure 1:**
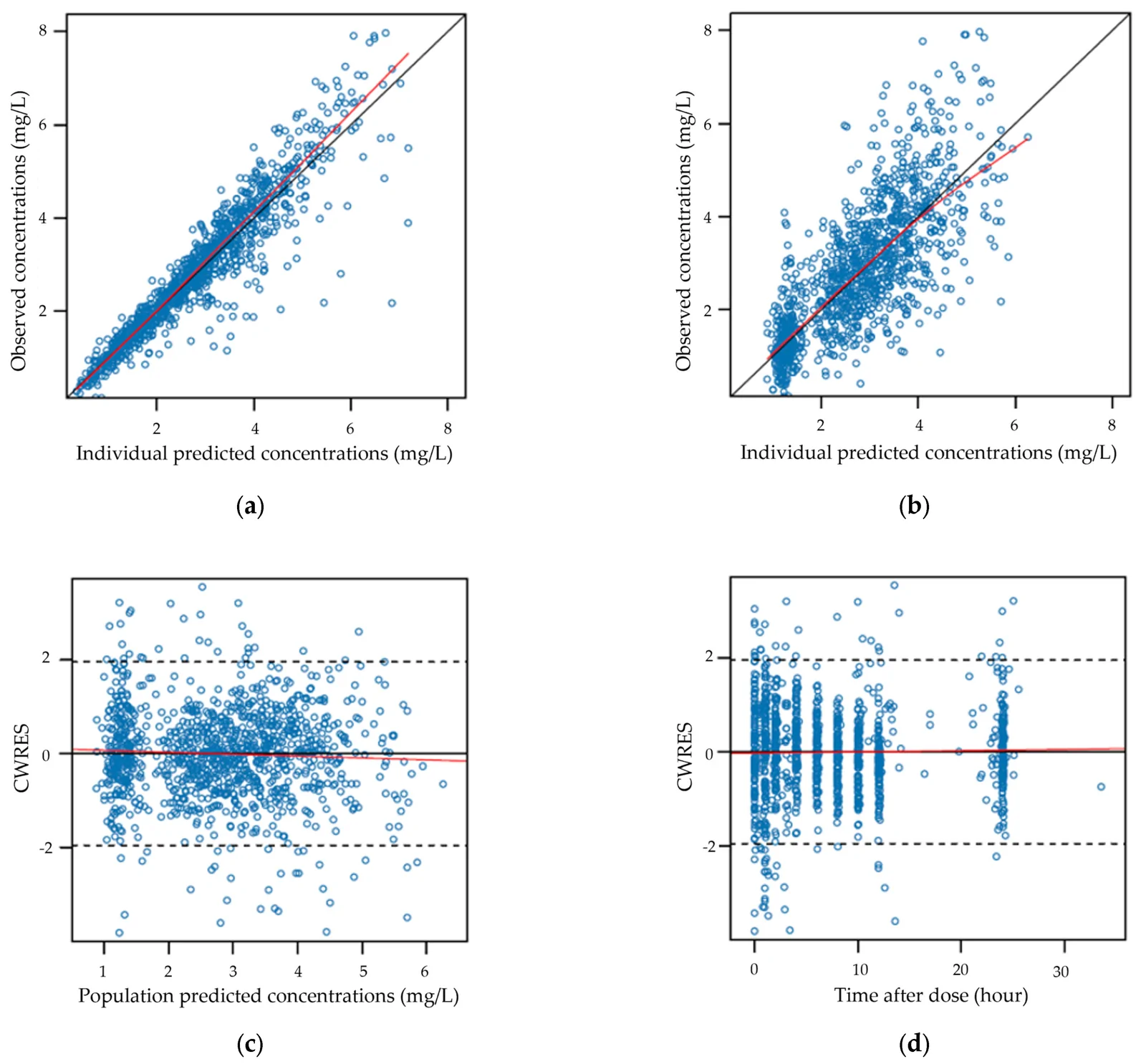
Goodness-of-fit plots of the final model of DTG: (**a**) observed DTG concentrations vs. individual predicted concentrations (IPRED); (**b**) observed DTG concentrations vs. population predicted concentrations (PRED); (**c**) conditional weighted residuals (CWRES) vs. PRED; (**d**) CWRES vs. time after dose.

The pharmacokinetics of DTG were best described by a one-compartment model with first-order absorption and elimination. A two-compartment model did not significantly improve the model fit. A combined proportional and additive error model was selected for the structural model as it significantly improved model fit over the proportional model (dOFV = −73.55, *p* < 0.0001). The inclusion of fixed allometric scaling significantly improved the base model fit (dOFV = −28.97, *p* < 0.0001). Models attempting to estimate these allometric exponents directly were evaluated but resulted in numerical instability and poor parameter precision; therefore, standard theoretical exponents were retained. To account for the delayed absorption of DTG, a lag-time was incorporated, which significantly reduced the objective function value (dOFV = −57.37). However, the IIV for lag time could not be accurately estimated and was therefore omitted from the final model. The bioavailability (F) was fixed to 1. The inclusion of IOV on the absorption-related parameters, including Ka, F, and ALAG1, did not result in significant improvement. The IIV was estimated for CL/F, V/F and F. The covariate analysis identified the Group 3 variant genotypes (UGT*1A1* poor metabolizer + *NR1I2* 63396 TT) as the only significant covariate influencing CL/F (dOFV = −8.28, *p* = 0.004). The estimated parameters of the final model are presented in Table 2. The CL/F of DTG was defined according to Equation (1):

(1) $$\frac{CL}{F}=0.835\times {\left(\frac{WT}{61}\right)}^{0.75}\times (1+\theta_{G3}\times G3)$$

where WT represents the BW (kg) of the individual, G3 denotes the Group 3 variant genotypes (UGT*1A1* poor metabolizer + *NR1I2* 63396 TT), which was assigned a value of 1 if present and 0 if absent. $\theta_{G3}$ represents the fractional change in CL/F associated with this genotype. Based on the final model, the estimated CL/F for Thai PLWH weighing 61 kg and not carrying Group 3 variant genotypes was 0.835 L/h. The DTG CL/F was 25.1% lower in PLWH carrying the Group 3 variant genotypes (UGT*1A1* poor metabolizer + *NR1I2* 63396 TT) compared to those PLWH who do not carry Group 3 variant genotypes. Furthermore, a 10 kg increase in BW was associated with a 12.06% increase in CL/F. The goodness-of-fit plots for the final model are presented in Figure 1.

### 3.2. Model Evaluation

Of 1000 bootstrap runs, 908 runs were minimized successfully with the successful covariance. The results of the bootstrap analysis are summarized in Table 2. The median parameter estimates and their 95% CIs obtained from the bootstrap analysis were in close agreement with the estimates from the final NONMEM model. These findings confirm the reliability of the parameter estimates of the final model from NONMEM.

**Figure 2:**
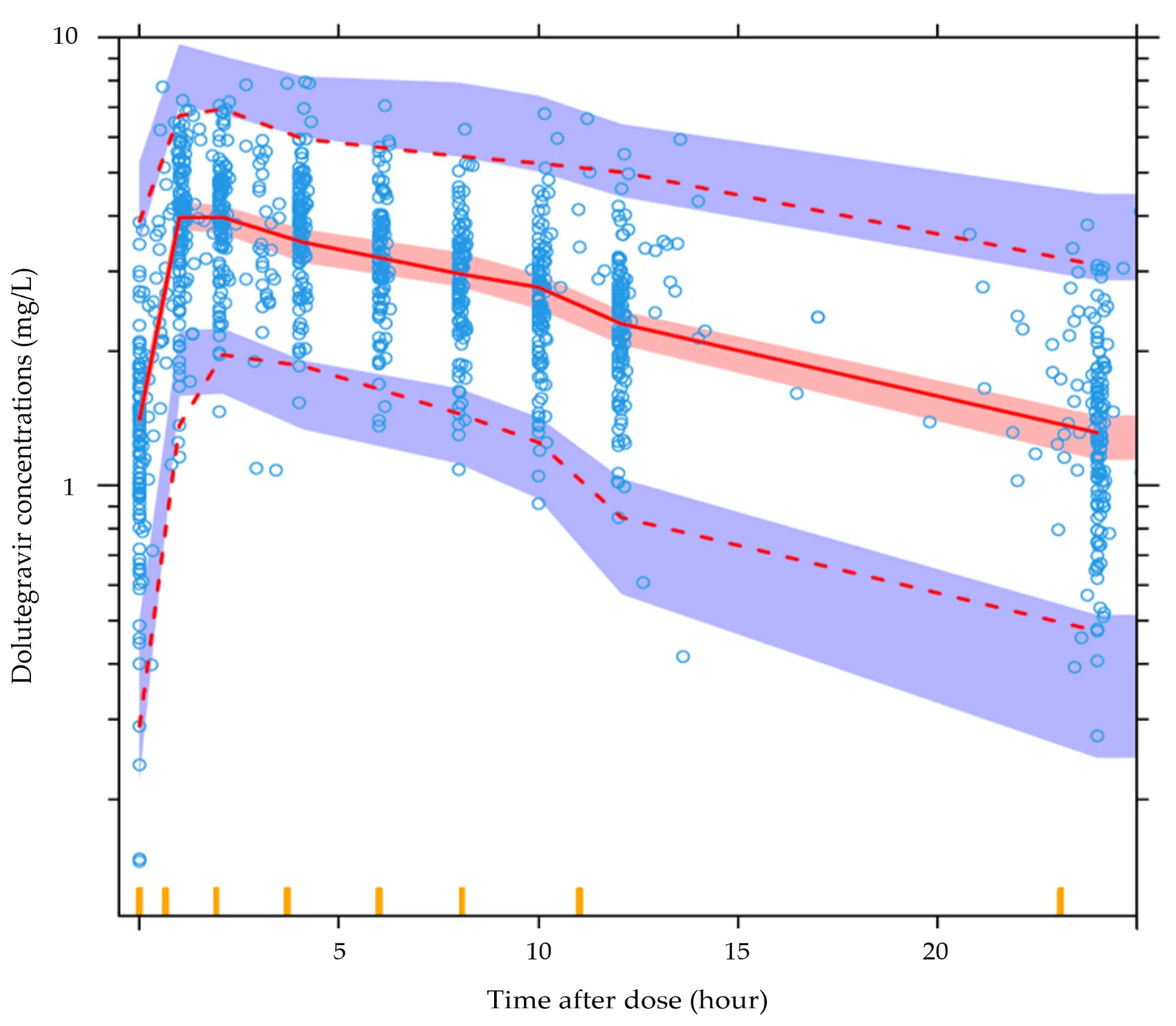
VPC plot of the final model. The solid line represents the 50th percentile of the observed concentrations, while the dotted lines denote the 5th and 95th percentiles of the observed concentrations. The shaded areas are the 95% CIs for the corresponding model-predicted percentiles. CI, confidence interval; DTG, dolutegravir; VPC, visual predictive check. (prediction interval).

The VPC plot obtained from 1000 simulations is shown in Figure 2. The observed medians, as well as 5th and 95th remained consistently within 95% CIs of the corresponding simulated values. These findings demonstrate that the final model accurately captures both the central tendency and the variability of the observed DTG concentrations. Consequently, the model was considered robust and suitable for subsequent dose optimization simulations.

### 3.3. Simulations for Dose Optimization

**Figure 3:**
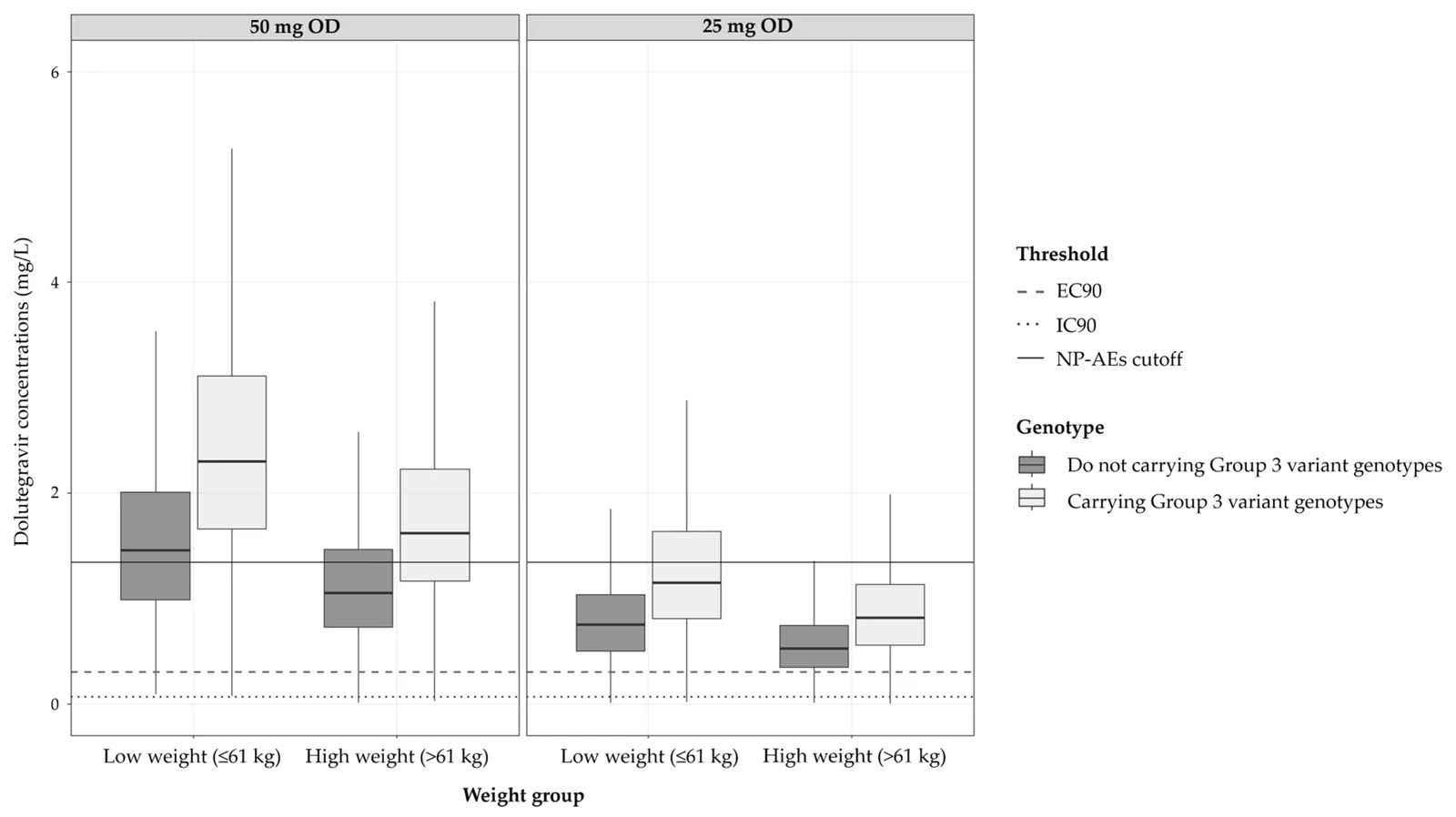
Box plots of model-simulated DTG trough concentrations following 50 mg and 25 mg OD regimens, stratified by low-weight (≤61.0 kg) and high-weight (>61.0 kg) groups. The boxes represent the 25th, 50th (median), and 75th percentiles, while the whiskers denote the 5th and 95th percentiles. Horizontal lines indicate the in vitro protein-adjusted IC90 (0.064 mg/L), the in vivo EC90 (0.3 mg/L), and the safety threshold for NP-AEs (1.34 mg/L).

Monte Carlo simulations for the standard dose of 50 mg OD revealed that over 95% of individuals had C_trough_ exceeding both the IC90 and EC90 thresholds across all BW and genetic subgroups. However, a substantial proportion of individuals (30.46% to 85.68%) that had DTG C_trough_ exceeded the safety threshold (>1.34 mg/L). This risk was particularly pronounced in the low-weight group with Group 3 variant genotypes, where approximately 85.68% of individuals reached concentrations associated with an increased risk of NP-AEs (Figure 3 and Table 3).

To evaluate potential dose optimization, a reduced dose of 25 mg OD was simulated. This regimen maintained an IC90 and EC90 attainment rate of >90%, across most subgroups. However, in the high-weight group of individuals who do not carry Group 3 variant genotypes, the EC90 attainment decreased to 80.91%. Regarding safety, the proportion of individuals exceeding the safety threshold (>1.34 mg/L) was substantially reduced to less than 20% for the majority of the population, although it remained at approximately 40% among the low-weight individuals who carried Group 3 variant genotypes (Table 3 and Figure 3).

## 4. Discussion

The present analysis substantially extends our previously published work, which evaluated intensive pharmacokinetic profiles via non-compartmental analysis in 104 Thai PLWH [31]. Here, the study population expanded to 221 participants (1122 plasma concentrations) through the integration of 117 additional individuals providing sparse pharmacokinetic data. Distinct from that prior descriptive study, this investigation leverages nonlinear mixed-effects modeling to quantify inter-individual and residual variability, identifies a significant multigenic interaction on DTG CL/F, and employs in silico Monte Carlo simulations to evaluate dose optimization strategies.

In our population PK analysis, DTG pharmacokinetics were best characterized by a one-compartment model with first-order absorption and elimination, incorporating a lag time. These results align with previous pharmacokinetic studies in both Thai and non-Asian (Caucasian) populations [9,13,14,15,32]. While previous studies have reported a two-compartment model for DTG [12,33], our data during the initial absorption phase (0–3 h) were relatively sparse, consisting of 79 observations from 79 individuals. Although our study design included intensive sampling at 1, 2, and 4 h post-dose, the overall data density was insufficient to support a more complex structural model.

UGT1A1 is a major enzyme responsible for DTG metabolism [16]. Thus, it is possible that DTG exposure may be influenced by *UGT1A1* polymorphisms. Previous observational studies have reported higher DTG exposure and a higher frequency of NP-AEs among individuals with reduced function UGT*1A1* phenotypes (intermediate or poor metabolizers), specifically those carrying the *UGT1A1* *28 or *UGT1A1* *6 alleles [20,31,34]. In our nonlinear mixed-effect analysis, however, individual UGT1A1 metabolizer phenotypes were not retained as independent covariates on DTG CL/F. The lack of statistical significance for individual *UGT1A1* polymorphisms on DTG CL/F might be attributed to the predominant effect of BW through allometric scaling, which potentially mask more modest genetic effects. Furthermore, the nature of population PK analysis, which explicitly partitions IIV, may have diminished the detectable impact of individual genes if their contribution to the overall variance was relatively small. Additionally, the low frequency of homozygous variant carriers in our cohort may have limited the statistical power to detect significant differences for single-gene effects.

Our findings highlight the influence of combined pharmacogenetic factors on DTG exposure. While individual *UGT1A1* polymorphisms alone were not independently impact DTG CL/F, a significant reduction in DTG CL/F (−25.1%) was observed among individuals carrying both the UGT*1A1* poor metabolizers phenotype and the *NR1I2* 63396 TT genotype. This supports a synergistic gene–gene interaction, consistent with previous reports [8], and underscores the importance of integrating multigenic profiles rather than single polymorphisms when predicting DTG pharmacokinetics. Elliot et al. found that the combined genotype of UGT*1A1* poor metabolizer and *NR1I2* 63396 TT was associated with significantly increased DTG exposure, including a 79% increase in the 24 h area under the concentration-time curve (AUC_0–24_) and a 47% increase in maximum concentration (C_max_) [8]. This synergistic relationship suggests that the interplay between the primary metabolic enzyme (UGT1A1) and its regulatory nuclear receptor (*NR1I2*) could be a critical determinant of DTG exposure. Consequently, PLWH carrying these combined variants are at a higher risk of increased drug exposure, which may necessitate closer clinical monitoring or preemptive dose adjustment.

Conversely, our results differ from those reported by Spector et al., which indicated that the *NR1I2* TT or CT genotypes independently led to a 25% reduction in DTG AUC_0–24_ within a pediatric population [35]. The *NR1I2* 63396 TT genotype is generally believed to enhance expression of the nuclear receptor PXR, which in turn upregulates key metabolic and transport pathways, including UGT1A1, CYP3A4, and BCRP, leading to reduced drug exposure [36,37]. However, when combined with the UGT*1A1* poor metabolizer phenotype, this regulatory effect appears to be altered. This discrepancy highlights the complexity of the PXR-mediated regulatory pathway. It is hypothesized that in the presence of compromised UGT1A1 function, the compensatory induction mechanism via PXR may be diminished or overridden. Despite these insights, the exact molecular interplay between these variants on DTG pharmacokinetics remains to be fully elucidated. Although individuals carrying the Group 3 variant genotypes accounted for 5.0% (n=11) of the study population, its incorporation on CL/F yielded a statistically significant reduction in objective function value (dOFV = −8.28, *p* = 0.004). Furthermore, the stability of this estimated 25.1% CL/F reduction was substantiated by non-parametric bootstrap resampling, which demonstrated close agreement with the final model estimate. Nonetheless, given the small sample size of this specific subgroup, these findings should be interpreted with appropriate caution, and the exact magnitude of this multigenic effect warrants further validation in larger prospective cohorts.

Consistent with several previous reports [31,38], we did not observe a significant association between the *ABCG2* 421C>A (rs2231142) polymorphism and DTG pharmacokinetics in our model. These results contrast with earlier findings, which demonstrated higher C_max_ of DTG in carriers of the variant A allele, particularly those with the homozygous AA genotype [8,22,35]. While *ABCG2* acts as an efflux transporter for DTG, the discrepancy between our findings and prior reports might stem from differences in study design. Those studies observed significant differences in C_max_ through intensive sampling, particularly during absorption, whereas our population PK approach relied primarily on sparse sampling, which may be less sensitive to transport-mediated changes during the absorption phase. Moreover, population-specific genetic backgrounds and limited statistical power may also contribute to this discrepancy among studies. Consequently, the role of the *ABCG2* 421C>A genotype in DTG disposition across diverse ethnic populations warrants further investigation in larger, well-powered studies.

Due to its high potency, a standard once-daily dose of 50 mg DTG yields a steady-state trough concentration (C_trough_) approximately 13-fold above the protein-adjusted, in vitro IC90 threshold of 0.064 mg/L [7]. Furthermore, a previous dose-ranging clinical study indicates that lower DTG doses, specifically 10 mg and 25 mg, achieve virological suppression comparable to the 50 mg regimen [27]. Consequently, a more conservative target of C_trough_ > 0.3 mg/L, which corresponds to the average minimum concentration observed with a 10 mg once-daily dose, has been advocated as the clinical in vivo 90% effective concentration (EC90) threshold. Elevated DTG exposure has been associated with NP-AEs, leading to DTG discontinuation [20], although a consensus on the definitive safety threshold for predicting NP-AEs has not yet been established. Yagura et al. reported that individuals with a median C_trough_ exceeding 1.34 mg/L experienced an increased risk of NP-AEs, while levels above 1.7 mg/L were specifically associated with treatment discontinuation [3,29]. In the present study, we adopted the more conservative 1.34 mg/L cutoff as our safety threshold to ensure a more stringent assessment of potential toxicity. Our simulations predicted that DTG exposure following the standard DTG dose (50 mg OD) in Thai PLWH remained consistently above both the IC90 and EC90 efficacy thresholds across all subgroups. However, this standard dose resulted in median C_trough_ exceeding the safety threshold, with the proportion of individuals at risk ranging from 30.46% in high-weight individuals who do not carry Group 3 variant genotypes to as high as 85.68% in low-weight individuals carrying Group 3 variant genotypes. These findings underscore the clinical relevance of both BW and pharmacogenetic factors in determining DTG exposure. However, it should be noted that this safety threshold was derived from a Japanese cohort and has not been prospectively validated in Thai PLWH; therefore, its application in the present analysis remains exploratory.

A reduced dose of DTG 25 mg OD was explored as a potential alternative for Thai PLWH to optimize the balance between efficacy and safety. Our results indicate that this lower dose provides sufficient C_trough_ to meet both the IC90 and EC90 efficacy thresholds in over 80–90% of the simulated population, while reducing the risk of exceeding the safety threshold and consequently the potential for NP-AEs. The greatest benefit was observed in individuals at highest risk, those with low BW and Group 3 variant genotypes. The proportion of having a median DTG C_trough_ above the 1.34 mg/L safety cutoff decreased from 53.39% to 12.01% in the low-weight group and from 30.46% to 2.69% in the high-weight group. Most notably, for the Group 3 variant genotypes, the percentage of individuals having median C_trough_ above the safety threshold decreased substantially from 85.68% to 39.61% in the low-weight group and from 66.00% to 13.90% in the high-weight group. These findings inferred that DTG 25 mg OD could be an appropriate dosage alternative for Thai PLWH experiencing or at high risk for DTG-related toxicities.

Despite these promising findings, implementation of reduced DTG dosing in routine clinical practice presents important challenges. In Thailand and many low-middle income settings, DTG is commonly administered as part of fixed-dose combinations (FDCs) with TAF/FTC or TDF/3TC. This limits the feasibility of dose adjustment without affecting the accompanying nucleoside reverse transcriptase inhibitors. Reducing the DTG component within these FDC formulations would also reduce exposure to the nucleoside reverse transcriptase inhibitor backbone, potentially compromising virological failure. Therefore, while our simulation results support the pharmacokinetic rationale for DTG dose reduction, clinical validation and formulation considerations are required to confirm long-term efficacy and safety outcomes.

These findings have implications in specific settings. During rifapentine-based tuberculosis preventive therapy (e.g., 1HP or 3HP), enzyme induction is known to reduce DTG exposure. However, individuals with higher baseline exposure, specifically those with low BW or high-risk genotypes, may achieve therapeutic levels without DTG dose escalation for 1 HP, though further confirmatory studies are warranted. Similarly, the known interaction with cation-containing products (e.g., calcium, iron, or antacids), which reduces DTG absorption, may be less clinically detrimental in this group. Collectively, these findings support a precision medicine approach that integrates BW and pharmacogenetic factors to optimize DTG use, particularly in resource-limited and ethnically diverse populations.

Our study had several limitations. First, although total bilirubin has been identified as a significant covariate impacting CL/F of DTG in previous studies [14,15], it was not investigated in our study due to substantial missing data, with only 16.29% of participants having total bilirubin records. Consequently, the potential competitive inhibition between total bilirubin and DTG metabolism via UGT1A1 could not be evaluated. Second, while the simulated 25 mg once daily regimen achieved adequate concentrations relative to the efficacy and safety targets, its clinical relevance and long-term safety require confirmation in prospective clinical trials. Third, the impact of food on DTG absorption could not be evaluated due to the lack of dietary data, despite evidence that food can increase the DTG AUC by 33–66% [39]. Lastly, while our sample size lacked sufficient statistical power to detect single-locus effects with small effect sizes, it possessed adequate power to identify the 25.1% clearance reduction observed in Group 3 variant genotypes. Furthermore, a sensitivity analysis re-fitting the model without these 11 subjects yielded similar structural PK parameters, indicating that baseline model parameters are robust (. Nonetheless, given the low prevalence of the Group 3 variant genotypes (5.0% of the cohort), these findings should be viewed as hypothesis-generating and warrant validation in larger, multi-ethnic cohorts. Moreover, other genetic polymorphisms, such as *CYP3A4*, *ABCB1*, and *SLC22A2*, were not investigated; however, their contributions to DTG pharmacokinetics are likely limited [16,22].

## 5. Conclusions

Our findings confirmed that BW and combined *UGT1A1* and *NR1I2* polymorphisms jointly influence dolutegravir disposition in Thai PLWH. Model-based simulations substantiated that a reduced 25 mg once-daily regimen maintains efficacy while markedly reducing the risk of exceeding concentrations linked to neuropsychiatric adverse events, particularly in individuals weighing ≤61.0 kg carrying combined UGT*1A1* poor metabolizer and *NR1I2* 63396 TT genotype.

Importantly, our results could be generalizable to broader global populations. Because CL/F was allometrically scaled with BW, the elevated exposure risk applies to lower-weight individuals across diverse ethnic and female cohorts. Furthermore, while specific allele frequencies vary geographically, the standardized CPIC poor-metabolizer phenotype unifies these variants. When considered alongside the widely distributed *NR1I2* 63396 TT variant, this multigenic interaction could serve as a useful conceptual basis for exploring high-exposure risks across diverse ethnic groups. Nonetheless, prospective studies in multi-ethnic cohorts are needed to confirm the broader generalizability of these findings.

## Figures and Tables

**Table 1:** Demographic characteristics of the study participants (*n* = 221).

Characteristic	Value
No. of participants of the following sex:	
Male, *n* (%)	119 (53.8)
Female, *n* (%)	102 (46.2)
Age (years), median (IQR) [min-max]	46.0 (25.0) [18.0–83.0]
Weight (kg), median (IQR) [min-max]	61.0 (15.1) [36.8–115.1]
Serum creatinine ^a^ (mg/dl), median (IQR) [min-max]	0.88 (0.33) [0.51–1.61]
ALT ^b^ (U/L), median (IQR) [min-max]	29.0 (20.0) [7.0–142.0]
Total bilirubin ^c^ (mg/dl), median (IQR) [min-max]	0.52 (0.18) [0.19–1.19]
UGT1A1*6 221 G>A (rs4148323) polymorphisms	
GG	190 (85.97)
GA	31 (14.03)
AA	0
*ABCG2* 421 C>A (rs2231142) polymorphisms	
CC	121 (54.75)
CA	84 (38.01)
AA	16 (7.24)
*NR1I2* 63396 C>T (rs2472677) polymorphisms	
CC	33 (14.93)
CT	108 (48.87)
TT	80 (36.20)
*UGT1A1* (TA)_n_ repeats polymorphisms	
TA6/TA6	134 (60.63)
TA6/TA7	64 (28.96)
TA7/TA7	15 (6.79)
TA7/TA8	7 (3.17)
TA5/TA6	1 (0.45)
UGT1A1 phenotypes based on CPIC guidelines	
Extensive metabolizer	113 (51.13)
Intermediate metabolizer	82 (37.10)
Poor metabolizer	26 (11.76)
Group 1 variant genotypes; UGT1A1 poor metabolizer + *ABCG* 421 AA + *NR1I2* 63396 TT	0
Group 2 variant genotypes; UGT1A1 poor metabolizer + *ABCG2* 421 AA	1 (0.5)
Group 3 variant genotypes; UGT1A1 poor metabolizer + *NR1I2* 63396 TT	11 (5.0)
Group 4 variant genotypes; *ABCG2* 421 AA + *NR1I2* 63396 TT	5 (2.3)

ALT, Alanine Aminotransferase; ^a^ Data are from 216 PLWH participants. ^b^ Data are from 167 PLWH participants. ^c^ Data are from 36 PLWH participants.

**Table 2:** The final parameter estimates and bootstrap results.

Parameter (Units)	NONMEM Point Estimate	%RSE	95% CI		Bootstrap Estimates		
					Median	95% CI	
CL/F (L/h)	0.84	2.50%	0.79	0.88	0.83	0.80	0.88
V/F (L)	16.00	2.90%	15.10	16.90	16.00	15.10	17.00
Ka (h^−1^)	3.28	15.50%	2.29	4.29	3.21	2.41	5.04
F	1	-	FIX	FIX	1	1	1
ALAG (h)	0.29	9.90%	0.24	0.35	0.29	0.20	0.33
CL-G_3_	−0.25	30.30%	−0.40	−0.10	−0.25	−0.39	−0.07
CL-WT	0.75	-	FIX	FIX	0.75	0.75	0.75
V-WT	1	-	FIX	FIX	1	1	1
Inter-individual variability (%CV)							
IIV-CL	24.40	11.30%	17.95	28.91	23.81	18.28	29.38
IIV-V	14.80	29.40%	5.74	21.56	14.69	5.58	22.31
IIV-F1	23.10	15.30%	14.40	28.82	22.41	15.42	29.07
Residual variability (%CV)							
RUV prop	15.91	7.40%	13.40	18.01	15.64	13.43	18.02
RUV Add (mg/L)	0.02	38.10%	0.01	0.04	0.02	0.01	0.04

Abbreviations: %RSE, relative standard error; RSE defined as: (SEestimate/estimate) x 100, where SE is standard error; CI, confidence interval; CL/F, apparent oral clearance; V/F, apparent volume of distribution; Ka, absorption rate constant; F, bioavailability; ALAG, lagged-time absorption; CL-G_3_, the influence of Group 3 variant genotypes on CL/F; CL-WT, the influence of weight on CL/F; V-WT, the influence of weight on V/F; IIV, interindividual variability; RUV, residual unexplained variability.

**Table 3:** Percentage of in silico simulated individuals (*n* = 80,000) achieving in vitro protein-adjusted 90% inhibitory concentration (IC90; 0.064 mg/L) and in vivo 90% effective concentration (EC90; 0.3 mg/L) target and exceeding the 1.34 mg/L safety threshold.

DTG Regimens	Subgroup Stratification		Efficacy Threshold		Safety Threshold
	Genotyping Status	BW	IC90	EC90	>1.34 mg/L
50 mg once daily (OD)	PLWH who do not carry Group 3 variant genotypes	≤61.0 kg	99.87	98.38	53.39
		>61.0 kg	99.94	97.32	30.46
	PLWH who carrying the Group 3 variant genotypes	≤61.0 kg	100.00	100.00	85.68
		>61.0 kg	100.00	99.59	66.00
25 mg once daily (OD)	PLWH who do not carry Group 3 variant genotypes	≤61.0 kg	99.29	90.24	12.01
		>61.0 kg	98.44	80.91	2.69
	PLWH who carrying the Group 3 variant genotypes	≤61.0 kg	99.94	98.44	39.61
		>61.0 kg	99.72	94.65	13.90

## Data Availability

The data that support the findings of this study are available on request from the corresponding author.

## Abbreviations

The following abbreviations are used in this manuscript:

DTG	Dolutegravir
PLWH	People living with HIV
PK	Pharmacokinetic
NP-AEs	Neuropsychiatric adverse events
OD	Once daily
PTA	Probability of target attainment
CL/F	Apparent clearance
UGT1A1	Uridine diphosphate glucuronosyltransferase family 1 member A1
NR1I2	Nuclear receptor subfamily 1 group I member 2
INSTI	Integrase transfer inhibitor
%CV	Percent coefficient of variation
CYP	Cytochrome P450
CPIC	Clinical Pharmacogenetics Implementation Consortium
BCRP	Breast cancer resistance protein
ABCG2	ATP binding cassette subfamily G member 2
PXR	Pregnane X receptor
BW	Body weight
IC90	in vitro protein-adjusted 90% inhibitory concentration
EC90	in vivo 90% effective concentration
HIV-NAT	The HIV Netherlands Australia Thailand Collaboration
1HP	Daily isoniazid/rifapentine regimen
3HP	Weekly isoniazid/rifapentine regimen
LTBI	Latent tuberculosis infection
ART	Antiretroviral therapy
TAF	Tenofovir alafenamide
TDF	Tenofovir disoproxil fumarate
FTC	Emtricitabine
3TC	Lamivudine
ALT	Alanine aminotransferase
LC-MS/MS	Liquid chromatography-tandem mass spectrometry
LLOQ	Lower limit of quantification
DNA	Deoxyribonucleic acid
PCR	Polymerase chain reaction
V/F	Apparent volume of distribution
IIV	Inter-individual variability
IOV	Inter-occasion variability
F	Bioavailability
Ka	Absorption rate constant
ALAG	Lag time
RUV	Residual unexplained variability
OFV	Objective function value
AIC	Akaike information criterion
GOF	Goodness-of-fit plot
%RSE	Percentage relative standard error
df	Degree of freedom
VPC	Visual predictive check
CI	Confidence interval
NONMEM	Nonlinear mixed-effect model
C_trough_	Trough concentration
AUC_0–24_	24 h area under the concentration-time curve
C_max_	Maximum concentration
FDCs	Fixed-dose combinations
